# Do active patients seek higher quality prenatal care?: A panel data analysis from Nairobi, Kenya

**DOI:** 10.1016/j.ypmed.2016.09.014

**Published:** 2016-11

**Authors:** Jessica Cohen, Ginger Golub, Margaret E. Kruk, Margaret McConnell

**Affiliations:** a Harvard T. H. Chan School of Public Health, 677 Huntington Ave, Boston, MA 02115, USA; b Jacaranda Health, Kiamumbi off Kamiti Road, P.O. Box 52595, 00100 Nairobi, Kenya

**Keywords:** Active patient, Prenatal care, Antenatal care, Quality of care, Kenya

## Abstract

Despite poverty and limited access to health care, evidence is growing that patients in low-income countries are taking a more active role in their selection of health care providers. Urban areas such as Nairobi, Kenya offer a rich context for studying these “active” patients because of the large number of heterogeneous providers available. We use a unique panel dataset from 2015 in which 402 pregnant women from peri-urban (the “slums” of) Nairobi, Kenya were interviewed three times over the course of their pregnancy and delivery, allowing us to follow women's care decisions and their perceptions of the quality of care they received. We define active antenatal care (ANC) patients as those women who switch ANC providers and explore the prevalence, characteristics and care-seeking behavior of these patients. We analyze whether active ANC patients appear to be seeking out higher quality facilities and whether they are more satisfied with their care. Women in our sample visit over 150 different public and private ANC facilities. Active patients are more educated and more likely to have high risk pregnancies, but have otherwise similar characteristics to non-active patients. We find that active patients are increasingly likely to pay for private care (despite public care being free) and to receive a higher quality of care over the course of their pregnancy. We find that active patients appear more satisfied with their care over the course of pregnancy, as they are increasingly likely to choose to deliver at the facility providing their ANC.

## Highlights

• Active prenatal care patients are prevalent in urban poor Nairobi, Kenya.
• Active patients are demographically similar to passive, but somewhat more educated.
• Active patients are increasingly likely to pay for private care over time.
• Over time, active patients are increasingly likely to view the care they receive as high quality.
• Over time, active patients are increasingly satisfied with the care they receive.

## 1 Introduction

Patients in the US and elsewhere are beginning to exercise more active choice in their selection of health providers (Leonard, 2013, Barbot, 2006, Hibbard and Greene, 2013). Increasingly, evidence is emerging that health outcomes and experiences with health care can be linked to how active and engaged patients are in managing their health care (Hibbard and Greene, 2013). The most commonly used tool for measuring a patient's level of health engagement—the “Patient Activation Measure” (PAM)—captures items related to, for example, one's belief in one's own responsibility for health care and the importance of taking an active role in one's own health, as well as beliefs about the importance of communicating with doctors and understanding the role of procedures and medications (Hibbard et al., 2005). Higher scores on this measure have been shown empirically to be linked to higher utilization of preventive health care, fewer delays in treatment seeking, and the seeking out of health information, including comparisons of provider quality (Hibbard and Greene, 2013, Hibbard, 2009, Fowles et al., 2009).

Patients in low income countries have often been characterized as “passive” with respect to health care (Kabakian-Khasholian et al., 2000). It has typically been assumed that poverty, limited or low-quality options for health care, and substantial information asymmetries between patients and providers lead to passive acceptance of whatever care is nearest and most affordable. However, increasing evidence is emerging that patients in developing countries may be more active than once believed. Leonard (2013) develops a model of the “active patient” in low-income countries and reviews the growing evidence that some low-income country patients are active. The active patient does not automatically seek care at the nearest and cheapest option but rather will incur costs (including travel time and out-of-pocket payments) to obtain higher quality health care and will actively seek to learn about provider quality (Leonard, 2013, Leonard et al., 2002, Leonard, 2007). In this framework, patients may become active because of a complex medical condition, because they perceive the care they would normally receive to be poor, or for some other reason. Strong evidence of active patients comes from the studies documenting high rates of “bypassing”—the phenomenon of avoiding nearby facilities and choosing more distant options that are perceived as providing more desirable care. Bypassing has been observed for primary care, obstetric care, male circumcision and other conditions (Leonard et al., 2002, Kruk et al., 2009, Ager and Pepper, 2005, Audo et al., 2005, Kruk et al., 2014, Golub et al., 2015). Prior evidence also shows that active patients seek out providers with better credentials when they have illnesses that are more complex to diagnose and treat (Leonard, 2007).

We explore antenatal care-seeking in Nairobi, Kenya, where pregnant women have a wide array of maternity facility options within close proximity. A large number of public, private and mission facilities offer antenatal care (ANC) and delivery services in Nairobi, with wide variation in the quality of services (National Coordinating Agency for Population and Development, Fotso and Mukiira, 2012, Ziraba et al., 2009). We define “active” ANC patients as women who switch providers over the course of their pregnancy and analyze the characteristics and health seeking behavior of these patients, exploring whether active patients appear to be seeking and receiving a different level of quality than “non-active” patients (i.e. those patients who stay with the same provider through pregnancy).

ANC is a key component (along with skilled obstetric care, family planning, etc.) of a package of services fundamental for maternal and newborn health (World Health Organization, 2010, Campbell and Graham, 2006). The WHO recommends that women receive at least four ANC appointments for a normal pregnancy, with the first visit initiated during the first trimester (WHO, 2007). Essential components of ANC appointments include (but are not limited to) monitoring of the progress of pregnancy and assessment of maternal and fetal wellbeing, detection of complications (e.g. hypertensive disorders and anemia), and anemia prevention (e.g. iron supplementation) (WHO, 2010). Both the technical quality of ANC and patient perceptions of the quality of care provided have been shown to vary both across and within African countries, with facilities often performing well along some dimensions of quality and not others (Duysburgh et al., 2013, Van Eijk et al., 2006, Ejigu et al., 2013, Boller et al., 2003, Hoque et al., 2008).

While several previous studies have explored the prevalence and care-seeking behavior of patients choosing a delivery facility, there has been little research on patient selection of clinics for preventive care, such as ANC. Much of the previous literature on active patients has also focused on rural rather than urban areas. Yet, urban areas such as Nairobi offer a particularly rich context for studying active patients because, given the large number of facilities available (and the wide variation in facility quality and type), pregnant women have more opportunities for active choice than women in rural areas.

We use a unique panel dataset in which over 400 pregnant women from peri-urban (the “slums” of) Nairobi were interviewed three times over the course of their pregnancy and delivery, allowing us to follow women's care decisions and their perceptions of the quality of care they received. We define active ANC patients as those women who switch ANC providers (i.e. those who attend ANC in more than one location) and explore the prevalence and characteristics of these patients. We analyze whether active ANC patients appear to be seeking out higher quality facilities and whether they are more satisfied with their care.

## 2 Methods

### 2.1 Study population

The study was conducted between February and September of 2015 in 24 peri-urban neighborhoods of Nairobi within Kiambu and Nairobi counties. These densely populated areas surrounding Nairobi are within 12–15 km of the city center and are primarily made up of low-income residential estates shared with industrial enterprises, especially in locations closer to the city center. These areas are characterized by a large number of public, private, and faith-based health facilities ranging from small pharmacies and outpatient care to large hospitals with maternity wards; these facilities also range widely in cost, size and services available. Study neighborhoods were selected based on: 1) the availability of both private and public facilities for ANC and delivery, 2) a composition of primarily lower-income residents, and 3) meeting a minimum level of security. Community health workers were engaged during the selection process to ensure these criteria were fulfilled. The fertility rate in Nairobi slums is 3.5 children per woman (APHRC, 2014) compared to the Nairobi provincial rate of 2.8 (KNBS, 2010).

### 2.2 Study procedures

Pregnant women (self-reported gestational age of 5, 6, or 7 months) aged 18 years and above were recruited through convenience sampling during a planned recruitment event within the study neighborhoods. During these events, field staff were stationed in community centers (e.g. markets and pedestrian intersections) where pedestrian traffic was high and where they could easily engage with interested community members and/or pregnant women passing through. Snowball sampling was used to supplement recruitment efforts: interested community members were asked if they knew pregnant women within the community who might be interested in the study and if so, they were encouraged to share the study flyer and/or come to speak with our field staff for more information. Eligible respondents were visited at their residence to obtain informed consent, ensure eligibility, and administer the baseline survey. The baseline survey occurred, on average, at 27.1 weeks gestation (median: 27.9; 95% CI: 26.7–27.5). A midline survey was administered at the respondent's home or work place and was scheduled to take place during her 8th month of pregnancy, occurring, on average, at 33.7 weeks gestation (median: 33.7; 95% CI: 33.5–33.9). A final survey was scheduled for 2–4 weeks post-partum and occurred, on average, at 3.5 weeks after delivery (median: 2.9; 95% CI: 3.3–3.7).

At the baseline visit, all women were asked about any ANC appointments they had for this pregnancy up until the day of the survey. For each subsequent survey, women were asked about any ANC appointments occurring since the last survey. For all ANC visits reported, confirmation of the visit and date was attempted in the ANC book, a small booklet provided at all Kenyan facilities documenting a patient's ANC history per pregnancy. 71.7% of visits were able to be confirmed in the ANC book, with no significant difference in ANC book confirmation between active and non-active patients (p = 0.626).

Prior to administering baseline, a sub-sample of women (n = 111) was randomly selected to be administered an abridged version of the baseline and midline surveys in case the extensive questions related to birth planning were found to influence behavior. Only one of our outcome variables is unavailable for the 27.6% of respondents given the abridged survey.

### 2.3 Sample

**Fig. 1:**
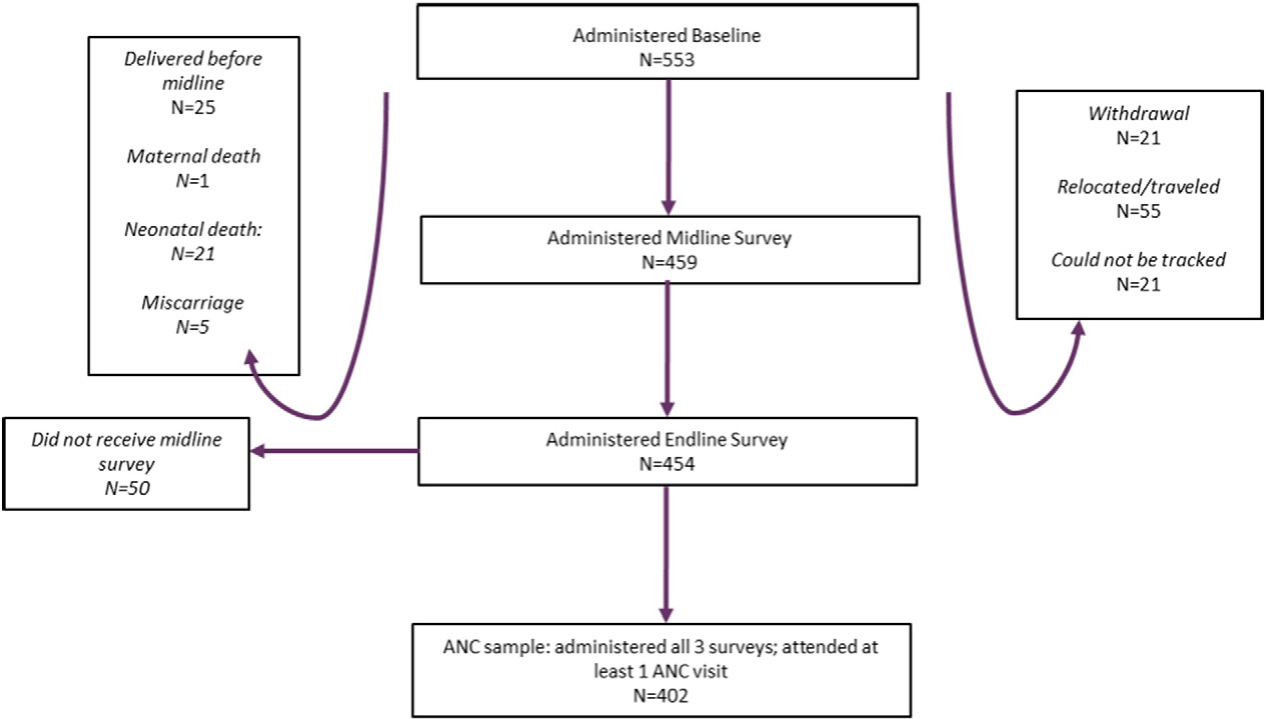
Survey samples and reasons for attrition.

A total of 553 women were surveyed at baseline, 459 at midline and 454 at endline (Fig. 1). Of the 553 surveyed at baseline, 21 women withdrew from the study, 21 could not be tracked for either the midline survey or endline survey or both, and 55 relocated out of the study area. Most relocations were to places outside of Nairobi and were temporary, as it is common to stay with family just before and after the birth of a child. A further 25 women delivered before midline, and there was one maternal death, 21 neonatal deaths and 5 miscarriages. 404 women were surveyed at all three visits. Among these, 2 had no ANC appointments and are dropped from the sample, leaving an analysis sample of 402 women. A total of 1621 ANC visits were reported on in our sample, occurring at a total of 165 different facilities.

### 2.4 Active patient definition

We define “active patients” as those women who switch ANC providers at least once during pregnancy. Of the 402 women in our sample, 139 (34.6%) were active patients.

### 2.5 Outcomes

Outcomes were derived from self-reported data (with the occurrence and date of ANC appointments confirmed in booklets 71.7% of the time) about ANC and delivery care collected during the three surveys. These included the frequency, timing, location, and quality of ANC visits, as well as the type of facility attended for ANC (private, public or other). An equally-weighted 6 point quality index was generated to capture whether respondents received various essential services during their visit and included the following components: whether the patients' weight was checked, blood pressure checked, fundal height measured, whether the baby's heart rate was measured, whether a urine sample was collected, and whether iron supplements were given. These measures were based on ANC quality measures captured in the 2014 Kenya Demographic and Health Survey and adapted to include baby's heart rate measurement and iron supplementation based on advice from maternal health experts in Kenya. We explore whether these services were conducted at each ANC visit. We also present results for each component of the index separately.

We construct two measures of patients' perceptions of the quality of care. First, we construct a binary variable equal to one if the respondent stated she would rate the quality of care she received as “excellent” and zero if she rated it “poor”, “fair” or “good”. The second measure is based on a question derived from a facility ranking exercise conducted with women during the baseline and midline surveys. In this exercise women were asked to list all of the facilities they were considering for delivery and were then asked to rank them relative to the other facilities being considered along a number of dimensions, including perceptions of overall quality. From this, we construct a binary variable equal to one if the woman ranked the facility she was using for ANC highest in terms of overall quality and zero otherwise.

Finally, we report on whether a woman ultimately delivered at the facility where she attended ANC, a measure of the patient's satisfaction with care. Since not all ANC facilities offer delivery services, we also report this outcome for the restricted sample of women attending ANC at facilities that offer delivery services.

In order to measure changes in the quality of care during pregnancy, we report outcomes for each of the first three ANC visits. 90% of women in our sample had at least three ANC visits, but only 66% had at least four visits. Since we want to demonstrate how care changes over the course of pregnancy for the majority of our sample—without confounding variation from large changes in sample composition—we restrict the analysis to the first three visits. Results for the fourth visit are presented in an appendix for comparison.

### 2.6 Statistical analyses

We test for differences in characteristics of active and non-active patients by running simple linear regressions of the variable of interest (demographic characteristics as well as characteristics of the pregnancy and delivery) on a binary variable for whether the patient is “active” and a constant term. We present the coefficient on the “active” variable, representing the difference in means between active and non-active patients, and the two-tailed p-value for the null hypothesis that the coefficient on “active” is equal to zero. We use the same approach to test for differences in our outcome measures related to quality of care, except that each regression is run for the first, second and third ANC visit separately. Since many of our outcome measures are binary and the quality index is an event count variable, to check the robustness of our results to specification choice we also present alternative specifications with logistic regression for binary outcomes and Poisson regression for the quality index in an appendix table. In all regressions, robust standard errors are clustered at the neighborhood level.

### 2.7 Ethical considerations

The protocol including all study materials was approved in the United States by Harvard T. H. Chan School of Public Health's Institutional Review Board and in Kenya by the Amref Health Africa (formerly, AMREF) Ethics and Scientific Review Committee.

## 3 Results

Table 1 presents demographic characteristics for the entire study sample (n = 402) and for active (n = 139) and non-active (n = 263) ANC patients separately. Women in our sample are on average 25.5 years old. Most women are married and have gone beyond primary school. Roughly two-thirds of the sample is multiparous.

Active and non-active patients appear similar along most of these demographic characteristics—including all measures of assets and personal monthly income—with the only significant difference in the level of educational attainment: active patients are significantly more likely to have gone to post-secondary school (p = 0.035). Appendix Table A1 shows that the 151 women who were surveyed at baseline but were not in the final analysis sample do not significantly differ from the analysis sample along any of these characteristics except for a small difference in age of 1.3 years (p = 0.004).

The health seeking behavior of active and non-active patients for ANC and delivery is presented in Table 2. Women in our sample have on average four ANC appointments throughout pregnancy and begin seeking ANC at roughly 19 weeks gestation. This is consistent with data from urban Kenya as a whole, where 60% of urban women attend at least four ANC visits and the median time of first visit is 5.6 months (DHS 2008–09). While active patients attend their first ANC appointment 2.8 weeks later in pregnancy than non-active patients (p < 0.001), they ultimately have an average of 0.7 more ANC visits (p < 0.001). While non-active patients (by construction) only visit one ANC facility, active patients visit 2.3 facilities on average (p < 0.001). Active patients are 7.6 percentage points more likely to have a high risk pregnancy (p = 0.036). A large difference between active and non-active patients is the utilization of private facilities for ANC: active patients are nearly six times as likely to have ever gone to ANC in a private facility (43.2% vs. 7.6%, p < 0.001). While active patients are 12% less likely to deliver in a public facility, this difference is just above conventional significance levels (p = 0.08). We find no significant differences in the likelihood of going for ANC or delivery outside of one's own neighborhood.

We now turn to how care-seeking and care quality over the course of pregnancy differ for active and non-active patients. The first three rows in Table 3 present differences in the types of facilities active patients visit over the course of pregnancy. While active patients were not significantly more likely to visit a private facility at the first visit (p = 0.113), by the third ANC visit they are 18.7% more likely to be using a private facility than non-active patients (p < 0.001). Active patients start out 17.7% less likely to be attending ANC at a facility in their own neighborhood (p = 0.005) but, by the third visit, are not less likely than non-active patients to be doing so (p = 0.113). Taken together, these results suggest active patients are seeking out private care closer to home, although the majority still use public facilities and travel outside their own neighborhood. More than a third of women attend ANC at facilities that do not offer delivery services, but the use of these facilities does not vary significantly between active and non-active patients.

The rest of the measures in Table 3 explore how ANC service quality, patient quality perceptions and satisfaction with care differ for active and non-active patients over the course of pregnancy. Facilities perform fairly well based on our 6-point service quality index at an average of 4.8 for active patients and 5.1 for non-active patients at the first visit. Active patients receive a modest, but significantly lower number of services at the first visit (− 0.34; p = 0.003), but this gap closes by the third visit. Notably, the gap is closing because services for non-active patients are getting worse, not because active patients are receiving more services. Table 4 breaks out each element of the quality index, showing that facilities perform best on weight and blood pressure measurement. While the likelihood of fundal height and fetal heart rate measurement increases over the pregnancy, urine testing and iron supplementation become less likely. Active patients are significantly less likely than non-active patients to receive each element of the quality index except iron supplements at the first visit. For all elements except fetal heart rate measurement, the gap between active and non-active closes to be insignificant by the third visit (and the gap does close somewhat for fetal heart rate measurement).

At the first visit, active patients have lower perceptions of the quality of care than non-active patients (Table 3). They are 11.4% less likely to rank their facility as highest quality among alternatives than non-active patients (p = 0.022) and they are somewhat, though not significantly so, less likely to feel the care was “excellent” (− 3.5%; p = 0.295). By the third visit, however, active patients are 12.1% more likely to feel the care was excellent (p = 0.012). Further, the probability that active patients rank the ANC facility they are using as highest quality increases from 5.7 at the first visit to 21.4 at the third visit, closing the gap with non-active patients so that they are no longer significantly different from one another at the third visit.

We consider whether or not a patient delivers at the place she attended for ANC to be a measure of her satisfaction with the quality of care provided. Only 7.2% of active patients end up delivering in the facility used for the first ANC appointment, relative to 24.5% of non-active patients (p = 0.001). By the third visit, this gap has closed so that, while the active patients are still somewhat less likely to deliver in the ANC facility, the difference is much smaller and is insignificant (− 5.3%; p = 0.355). Since not all ANC facilities offer delivery services, it could be that ANC patients are increasingly likely to deliver at the place they are attending for ANC not because of increased satisfaction with care but because of an increased likelihood of attending ANC at a facility that performs delivery. To explore this possibility, in the last row of Table 3 we present the fraction of women delivering at the facility used for ANC among those attending ANC at a facility that offers delivery. Among active patients in this sample, the likelihood of delivering in the ANC facility increases from 13.5% at the first visit to 30.3% at the last visit, again closing a gap with non-active patients that is large and significant at the first visit. This suggests that the increased likelihood of delivering at the ANC facility among active patients is indicative of increasing satisfaction with the care they are receiving at the facilities they attended later in pregnancy.

We explore the robustness of these results in several appendix tables. Table A2, Table A3 demonstrate that the results in Table 3 do not change meaningfully when we control for the demographic characteristics in Table 1 or when we restrict the sample to women who do not have high risk pregnancies, respectively, suggesting that our results are not driven by differential characteristics of active patients. In Appendix Table A4, we present our main outcomes using logistic regression for all binary dependent variables (results are in odds ratios) and Poisson regression (results are in incidence rate ratios) for the quality index specification and find similar results to Table 3. Finally, in Appendix Table A5, we present all of the outcomes in Table 3 for the fourth ANC visit as well. As noted above, our main results do not include the fourth visit because 40% of women do not have a fourth visit and, thus, the sample changes substantially between the third and fourth visit. However, we do not find any big changes in outcomes between the third and fourth visit, suggesting that the focus on the first three visits in our main analysis is not misleading of how outcomes evolve for active vs. non-active patients over the course of the pregnancy.

## 4 Discussion

We find evidence that a substantial share of pregnant women in peri-urban Nairobi are active patients in their choice of ANC providers, switching ANC facilities in what appears to be pursuit of providers offering higher perceived quality of care. While important known benefits stem from continuity of maternity care (Kerber et al., 2007), if active patients are switching to higher quality care this can confer health benefits as well. We cannot say in this analysis whether active patients are actually receiving higher quality care or are experiencing health benefits from their switch, but this is the focus of ongoing research. Active patients turn increasingly to private care, even though ANC in public facilities is free in Kenya, demonstrating an increasing willingness to pay for care over the course of pregnancy. We do not find strong evidence that active patients are of relatively higher socioeconomic status, suggesting that their use of private care is unlikely attributable to a higher ability to pay for it. The fact that active patients are more likely to have high-risk pregnancies is consistent with Leonard's (2013) model of the active patient, which describes care seeking as more active when one's condition is more complex, although our results do not change when we restrict our sample to women who are not high risk.

We find a fairly high level of quality with respect to services received during ANC, without much variation between active and non-active patients, but more variation in the patients' perceptions of the overall quality of care. Active patients' perceptions of the quality of care they are receiving start out lower than non-active patients and increase over the pregnancy, suggesting that they are searching for better providers. Toward the end of pregnancy, active patients appear happier with their care than non-active patients, but we cannot specify which aspects of quality are driving this increase in satisfaction. We do not see evidence that active patients receive a higher level of technical quality than their non-active counterparts. This could be because our survey tools do not capture important aspects of technical quality or because the level of technical quality we find is fairly high overall, leaving little room for improvement. It could also be that active patients' perceptions of overall care are more reflective of other, non-technical, dimensions of care (e.g. friendliness and respectfulness of staff, facility amenities, etc.). This is consistent with other research from the Nairobi slums, which demonstrates that women perceive the quality of care to be highest at the small, private (often unlicensed) maternity facilities, which are often unequipped technically to provide safe deliveries but which offer kinder, more respectful care and more comfortable amenities (Fotso and Mukiira, 2012).

We find evidence that active patients are increasingly satisfied with the ANC care they are receiving over the course of the pregnancy, as measured by an increasing likelihood of delivering at the facility providing their ANC. On the other hand, the majority of women in our sample are not delivering at their ANC facility, suggesting that the criteria being used to select an ANC facility and to select a delivery facility may be compartmentalized. Ongoing research is exploring why so few women deliver in their ANC facility and what criteria drive utilization of maternity facilities.

### 4.1 Study limitations

This paper documents the prevalence and characteristics of active patients among pregnant women in Nairobi and provides evidence that they are receiving higher perceived quality of care. However, we cannot say whether it is one thing or many that make them “active”. It could be due to a character trait, to a bad patient-provider match, to high risk conditions, to more informed social networks or some combination of these, and may differ across patients. Some of the frequent switching we see in this population could be driven by the occasional opening and closing of small delivery facilities. While we are not aware of any facilities in our study area opening or closing during the study period, the nurses at one commonly-used facility did go on strike for several weeks during our endline survey and this could have caused some women to change their delivery location. Our index of received services is short, reflecting the relatively simple nature of ANC care, and the services are widely provided in this setting. This limits our ability to discern differences in the quality of these services received between active and passive patients and we cannot say exactly what it is about providers that is causing women to switch. Our recruitment methods relied on convenience and snowball sampling, which may have led to overrepresentation of women with more initiative and possibly a higher concentration of active patients than would be found based in a representative sample. Our indices were generated from self-reports, which may be biased if women do not accurately remember or understand the care they received. We also do not have information on women's social networks, which may be important determinants of health care decisions in this population. Finally, our inference is limited to an urban slum population with many facility choices. Our results may not be representative of women in rural areas or even in non-slum regions of Nairobi.

## 5 Conclusions

Overall, we find that a substantial proportion of urban poor women are active health care consumers. Active patients are somewhat more educated but otherwise resemble non-active patients in their demographics. Switching ANC facilities may be a means of searching for a delivery facility: women try out different providers and settings for a routine, low acuity service to assist selection for a higher acuity health event. Women may also be switching in response to high risk conditions or because of a poor patient-provider match. More research is needed to understand how women choose ANC and delivery facilities in order to steer women to high quality care to improve maternal and newborn outcomes.

The following are the supplementary data related to this article.

Table A1 Demographic characteristics of analysis sample and attrition sample. Table A1

Table A2 Differences between active and non-active patients in type of ANC facility utilized and quality of care received by ANC visit number, adjusted for demographic characteristics. Table A2

Table A3 Differences between active and non-active patients in type of ANC facility utilized and quality of care received by ANC visit number among non-high risk patients. Table A3

Table A4 Differences in odds ratios and incidence rate ratios between active and non-active patients in type of ANC facility utilized and quality of care received by ANC visit number. Table A4

Table A5 Differences between active and non-active patients in type of ANC facility utilized and quality of care received by ANC visit number (including fourth ANC visit). Table A5

## Conflict of interest

The authors declare there is no conflict of interest.

## Transparency document

Transparency document. Image 1

## Figures and Tables

**Table 1:** Demographic characteristics of study sample and active patients, with clustered standard errors by neighborhood.

Characteristic	Total (n = 402)	Active (n = 139)	Non-active (n = 263)	Difference: active - non-active	[p-Value] Ho: difference = 0
	Mean, %	Mean, %	Mean, %		
Age (years)	25.5	25.0	25.8	− 0.8	[0.027]⁎⁎
Married/partnered	87.8%	90.6%	86.3%	0.043	[0.293]⁎⁎⁎
Multiparous	67.2%	65.5%	68.1%	− 0.026	[0.596]
Educational achievement					
Primary school or less	31.6%	27.3%	33.8%	− 0.065	[0.215]
Some level of secondary school	51.2%	49.6%	52.1%	− 0.025	[0.676]
Post-secondary school	17.2%	23.0%	14.1%	0.090	[0.035]⁎⁎
Employed	33.1%	29.5%	35.0%	− 0.055	[0.318]
Personal monthly income (USD)	48.8	54.3	45.8	8.5	[0.520]
Improved water source	88.8%	90.6%	87.8%	0.028	[0.430]
Improved toilet	88.3%	85.6%	89.7%	− 0.041	[0.288]
Owns mobile phone	91.3%	89.9%	92.0%	− 0.021	[0.550]
Has electricity	92.3%	90.6%	93.2%	− 0.025	[0.426]
Has television	75.9%	71.9%	77.9%	− 0.060	[0.122]
Has radio	77.6%	77.0%	77.9%	− 0.010	[0.804]

“Active” is defined as those who attended more than one ANC facility. Study took place in Nairobi, Kenya in 2015.

p-Values are from ordinary least squares regressions with the dependent variable indicated in Column 1 regressed on a constant term and a binary variable for “Active” and test whether the coefficient on “Active” is significantly different from zero.

Robust standard errors are adjusted for clustering at the neighborhood level.

There are 12 missing values for personal monthly income (5 active, 7 non-active respondents) who were unable or refused to estimate.

⁎⁎⁎ p < 0.01.

⁎⁎ p < 0.05.

**Table 2:** ANC attendance and delivery locations for active vs. non-active patients, with clustered standard errors by neighborhood.

Characteristic	Total (n = 402)	Active (n = 139)	Non-Active (n = 263)	Difference	p-Values
	Mean, %	Mean, %	Mean, %	Active - non-active	Active vs. nonactive
*ANC visits*					
Total ANC visits attended for this pregnancy (mean)	4.0	4.5	3.8	0.70	[< 0.001]⁎⁎⁎
Gestational age (weeks) at first ANC visit (mean)	19.3	21.1	18.3	2.80	[< 0.001]⁎⁎⁎
Number of ANC locations visited (mean)	1.4	2.3	1.0	1.30	[< 0.001]⁎⁎⁎
Ever been told she has a high risk pregnancy during this pregnancy	15.2%	20.1%	12.5%	7.60%	[0.036]⁎⁎
Ever attended ANC at private facility	19.9%	43.2%	7.6%	35.60%	[< 0.001]⁎⁎⁎
Percentage of ANC visits attended within neighborhood	15.9%	18.1%	14.7%	3.4%	[0.294]

*Delivery facility*					
Total number of facilities considered for delivery throughout pregnancy	3.460	3.547	3.414	0.132	[0.301]
Delivered in a facility within neighborhood	16.8%	14.0%	18.2%	− 4.2%	[0.236]
Delivered at public facility	66.2%	58.3%	70.3%	− 12.00%	[0.081]⁎

“Active” is defined as those who attended more than one ANC facility. Study took place in Nairobi, Kenya in 2015. p-Values are from ordinary least squares regressions with the dependent variable indicated in Column 1 regressed on a constant term and a binary variable for “Active” and test whether the coefficient on “Active” is significantly different from zero.

Robust standard errors are adjusted for clustering at the neighborhood level.

There are 8 missing values for gestational age at first ANC visit (4 active, 4 non-active) because some women did not know their estimated date of delivery; there are 8 missing values for delivering in a facility within neighborhood because 8 women did not deliver at a facility (3 active, 5 non-active).

⁎⁎⁎ p < 0.01.

⁎⁎ p < 0.05.

⁎ p < 0.10.

**Table 3:** Differences between active and non-active patients in type of ANC facility utilized and quality of care received by ANC visit number.

	First ANC visit (n = 402)				Second ANC visit (n = 398)				Third ANC visit (n = 364)			
	Active (n = 139)	Non-active (n = 263)	Difference: active - non-active	[p-Value] Ho: difference = 0	Active (n = 139)	Non-active (n = 259)	Difference: active - non-active	[p-Value] Ho: difference = 0	Active (n = 136)	Non-active (n = 228)	Difference: active - non-active	[p-Value] Ho: difference = 0
*Facility characteristics*												
Visit was at a private ANC facility	12.2%	7.6%	4.6%	[0.113]	20.9%	7.7%	13.1%	[< 0.001]⁎⁎⁎	25.7%	7.0%	18.7%	[< 0.001]⁎⁎⁎
Visit was at a facility in own neighborhood	23.0%	40.7%	− 17.7%	[0.005]⁎⁎⁎	30%	40.9%	− 10.7%	[0.101]	30.1%	40.8%	− 10.6%	[0.113]
Visit was at a facility that offers delivery services	52.6%	62.7%	− 10.2%	[0.143]	59.0%	62.5%	− 3.6%	[0.620]	64.9%	62.7%	2.2%	[0.687]

*Facility quality measures*												
Visit included (index out of 6): *weight, blood pressure, fundal height, baby heart rate measured, urine sample taken, iron supplements*	4.812	5.153	− 0.342	[0.003]⁎⁎⁎	4.481	4.614	− 0.132	[0.261]	4.585	4.586	− 0.001	[0.994]
Respondent rated the overall quality of ANC at this visit “excellent”	14.4%	17.9%	− 3.5%	[0.295]	25.9%	15.1%	10.8%	[0.010]⁎⁎⁎	25.7%	13.7%	12.1%	[0.012]⁎⁎
Respondent ranked facility used for this visit as highest quality	5.7%	17.1%	− 11.4%	[0.022]⁎⁎	11.4%	16.4%	− 5.0%	[0.315]	21.4%	17.4%	4.0%	[0.463]
Patient delivered at ANC facility used for this visit (all)	7.2%	24.5%	− 17.3%	[0.001]⁎⁎⁎	10.8%	24.1%	− 13.3%	[0.012]⁎⁎	18.4%	23.7%	− 5.3%	[0.355]
Patient delivered at ANC facility used for this visit (*among those attending ANC at a facility that offers delivery*)	13.5%	38.8%	− 25.3%	[< 0.001]⁎⁎⁎	18.3%	38.3%	− 20.0%	[0.002]⁎⁎⁎	30.3%	37.8%	− 7.4%	[0.361]

“Active” is defined as those who attended more than one ANC facility. Study took place in Nairobi, Kenya in 2015.

A total of 10 values for the 6-point quality index are missing because at least 1 value within the index was missing.

p-Values are from ordinary least squares regressions with the dependent variable indicated in Column 1 regressed on a constant term and a binary variable for “Active” and test whether the coefficient on “Active” is significantly different from zero, separately for each visit number.

Robust standard errors are adjusted for clustering at the neighborhood level.

The variable for ANC facility utilized ranked as highest quality was only asked to respondents who received the full baseline and midline surveys (first visit: n = 291; second visit: n = 287; third visit: n = 263).

Missing values across all 3 visits per variable: quality index (9); excellent services (1); ANC facility offers delivery services (4); delivery facility at facility utilized for ANC (4); delivered at ANC facility that offers delivery (4).

⁎⁎⁎ p < 0.01.

⁎⁎ p < 0.05.

**Table 4:** Differences between active and non-active patients in quality of care received broken out by each component by ANC visit number.

	First ANC visit (n = 402)				Second ANC visit (n = 398)				Third ANC visit (n = 364)			
Facility quality measures	Active (n = 139)	Non-active (n = 263)	Difference: active - non-active	[p-Value] Ho: difference = 0	Active (n = 139)	Non-active (n = 259)	Difference: active - non-active	[p-Value] Ho: difference = 0	Active (n = 136)	Non-active (n = 288)	Difference: active - non-active	[p-Value] Ho: difference = 0
ANC visit included												
Weight measured	0.942	0.985	− 0.042	[0.061]*	0.986	0.977	0.009	[0.526]	0.971	0.991	− 0.021	[0.254]
Blood pressure taken	0.928	0.977	− 0.050	[0.013]⁎⁎	0.935	0.965	− 0.030	[0.166]	0.941	0.965	− 0.024	[0.229]
Fundal height measured	0.734	0.820	− 0.086	[0.084]*	0.860	0.946	− 0.086	[0.039]⁎⁎	0.941	0.965	− 0.024	[0.311]
Baby heart rate measured	0.748	0.824	− 0.076	[0.029]⁎⁎	0.891	0.961	− 0.071	[0.016]⁎⁎	0.956	0.987	− 0.031	[0.126]
Urine sample	0.755	0.863	− 0.108	[0.032]⁎⁎	0.261	0.166	0.095	[0.054]⁎	0.163	0.114	0.049	[0.140]
Iron supplements given	0.698	0.688	0.010	[0.745]	0.543	0.598	− 0.055	[0.376]	0.559	0.548	0.011	[0.851]

“Active” is defined as those who attended more than one ANC facility. Study took place in Nairobi, Kenya in 2015.

p-Values are from ordinary least squares regressions with the dependent variable indicated in Column 1 regressed on a constant term and a binary variable for “Active” and test whether the coefficient on “Active” is significantly different from zero, separately for each visit number.

Robust standard errors are adjusted for clustering at the neighborhood level.

Missing values across all 3 visits include: blood pressure (1); fundal height (6); baby heart rate (4); urine (2); iron (1).

⁎⁎⁎ p < 0.01.

⁎⁎ p < 0.05.

⁎ p < 0.10.
