# Supplementary material for: Do active patients seek higher quality prenatal care?: A panel data analysis from Nairobi, Kenya
Source: Prev Med. 2016 Nov;92:74–81. doi: 10.1016/j.ypmed.2016.09.014 (PMC5100690; doi:10.1016/j.ypmed.2016.09.014)
Supplement: Table A1 — Demographic characteristics of analysis sample and attrition sample. [file mmc1.pdf]

Table A1. Demographic Characteristics of Analysis Sample and Attrition Sample

Characteristic	*ANC Analysis Sample (n=402)*	*Attrition Sample [Lost, Relocated, Death, Miscarriage] (n=151)*	*[p-value] Ho: Difference = 0*
	mean, %	mean, %	
**Age (years)**	25.5	24.6	[0.017]**
**Married/Partnered**	87.8%	90.1%	[0.475]
**Multiparous**	67.2%	63.6%	[0.246]
**Educational achievement**			
Primary school or less	31.6%	36.4%	[0.351]
Some level of secondary school	51.2%	45.7%	[0.153]
Post-secondary school	17.2%	17.9%	[0.847]
**Employed**	33.1%	32.0%	[0.778]
**Personal Monthly Income (USD)**	4,485	4,408	[0.926]
**Improved water source**	88.8%	93.9%	[0.185]
**Improved toilet**	88.3%	88.6%	[0.939]
**Owns Mobile Phone**	91.3%	88.7%	[0.313]
**Has Electricity**	92.3%	92.7%	[0.870]
**Has Television**	75.9%	70.7%	[0.316]
**Has Radio**	77.6%	76.7%	[0.779]

*** p<0.01, ** p<0.05

P-values are from ordinary least squares regressions with the dependent variable indicated in Column 1 regressed on a constant term and a binary variable for "In ANC Analysis Sample" and test whether the coefficient on "in ANC Analysis Sample" is significantly different from zero.

Robust standard errors are adjusted for clustering at the neighborhood level.

In the ANC sample, there are 12 missing values for personal income; in the attrition sample there are 4 missing values for personal income, 2 for improved water source, and 1 for improved toilet source
