# Supplementary material for: Do active patients seek higher quality prenatal care?: A panel data analysis from Nairobi, Kenya
Source: Prev Med. 2016 Nov;92:74–81. doi: 10.1016/j.ypmed.2016.09.014 (PMC5100690; doi:10.1016/j.ypmed.2016.09.014)
Supplement: Table A2 — Differences between active and non-active patients in type of ANC facility utilized and quality of care received by ANC visit number, adjusted for demographic characteristics. [file mmc2.pdf]

Table A2. Differences between Active and Non-Active Patients in Type of ANC Facility Utilized and Quality of Care Received by ANC Visit Number, Adjusted for Demographic Characteristics

	First ANC Visit (n=402)		Second ANC Visit (n=398)		Third ANC Visit (n=364)	
	Difference: *Active - Non-Active*	*[p-value] Ho: Difference = 0*	Difference: *Active - Non-Active*	*[p-value] Ho: Difference = 0*	Difference: *Active - Non-Active*	*[p-value] Ho: Difference = 0*
Facility Characteristics:						
Visit was at a Private ANC Facility	0.056	[0.137]	0.120	[0.002]***	0.175	[0.001]***
Visit was at a Facility in Own Neighborhood	-0.082	[0.596]	0.057	[0.172]	0.020	[0.690]
Visit was at a Facility that Offers Delivery Services	-0.128	[0.037]**	-0.077	[0.269]	-0.021	[0.658]
Facility Quality Measures:						
Visit Included (Index out of 6): *Weight, Blood Pressure, Fundal Height, Baby Heart Rate Measured, Urine Sample Taken, Iron supplements*	-0.257	[0.045]**	-0.146	[0.183]	-0.065	[0.522]
Respondent Rated the Overall Quality of ANC at this Visit "Excellent"	-0.025	[0.539]	0.100	[0.012]**	0.143	[0.004]***
Respondent Ranked Facility Used for this Visit as Highest Quality	-0.126	[0.016]**	-0.065	[0.164]	-0.010	[0.826]
Patient Delivered at ANC Facility Used for this Visit	-0.183	[0.001]***	-0.143	[0.011]**	-0.064	[0.226]
Patient Delivered at ANC Facility Used for this Visit (*Among those Attending ANC at a Facility that Offers Delivery*)	-28.3%	[0.001]***	-20.7%	[0.019]**	-8.0%	[0.376]

*** p<0.01, ** p<0.05

"Active" is defined as those who attended more than one ANC facility

P-values are from ordinary least squares regressions with the dependent variable indicated in Column 1 regressed on a constant term and a binary variable for "Active" and test whether the coefficient on "Active" is significantly different from zero, separately for each visit number.

Regressions control for age, marital status, parity, educational achievement, employment status, personal income, improved water source, improved toilet, and assets including phone, electricity, television and radio, and neighborhood of residence

Robust standard errors are adjusted for clustering at the neighborhood level.

The variable for ANC facility utilized ranked as highest quality was only asked to respondents who received the full baseline and midline surveys (First visit: n=291; Second visit: n=287; Third visit: n=263)

Missing values across all 3 visits (n=1164) per variable: ANC at private facility (35); ANC facility in neighborhood (35); quality index (44); excellent services (36); ANC facility highest quality ranking (23); ANC facility offers delivery services (39); delivery facility at facility utilized for ANC (37); delivered at ANC facility that offers delivery (37)
