# Supplementary material for: Do active patients seek higher quality prenatal care?: A panel data analysis from Nairobi, Kenya
Source: Prev Med. 2016 Nov;92:74–81. doi: 10.1016/j.ypmed.2016.09.014 (PMC5100690; doi:10.1016/j.ypmed.2016.09.014)
Supplement: Table A4 — Differences in odds ratios and incidence rate ratios between active and non-active patients in type of ANC facility utilized and quality of care received by ANC visit number. [file mmc4.pdf]

Table A4. Differences in Odds Ratios and Incidence Rate Ratios between Active and Non-Active Patients in Type of ANC Facility Utilized and Quality of Care Received by ANC Visit Number

	First ANC Visit (n=402)		Second ANC Visit (n=398)		Third ANC Visit (n=364)	
	Difference: *Active - Non-Active*	*[p-value] Ho: Difference = 0*	Difference: *Active - Non-Active*	*[p-value] Ho: Difference = 0*	Difference: *Active - Non-Active*	*[p-value] Ho: Difference = 0*
Facility Characteristics:						
Visit was at a Private ANC Facility	1.693	[0.097]*	3.150	[0.000]***	4.592	[0.000]***
Visit was at a Facility in Own Neighborhood	0.436	[0.000]***	0.625	[0.081]*	0.626	[0.097]*
Visit was at a Facility that Offers Delivery Services	0.658	[0.144]	0.861	[0.618]	1.100	[0.680]
Facility Quality Measures:						
Visit Included (Index out of 6): *Weight, Blood Pressure, Fundal Height, Baby Heart Rate Measured, Urine Sample Taken, Iron supplements*	0.934	[0.001]***	0.971	[0.251]	1.000	[0.994]
Respondent Rated the Overall Quality of ANC at this Visit "Excellent"	0.772	[0.292]	1.972	[0.002]***	2.191	[0.004]***
Respondent Ranked Facility Used for this Visit as Highest Quality	0.294	[0.013]**	0.658	[0.302]	1.290	[0.452]
Patient Delivered at ANC Facility Used for this Visit	0.239	[0.000]***	0.380	[0.005]***	0.726	[0.348]
Patient Delivered at ANC Facility Used for this Visit (*Among those Attending ANC at a Facility that Offers Delivery*)	0.247	[0.000]***	0.361	[0.002]***	0.718	[0.360]

*** p<0.01, ** p<0.05

"Active" is defined as those who attended more than one ANC facility

P-values are from logistic regressions with the dependent variable indicated in Column 1 regressed on a binary variable for "Active" and test whether the coefficient on "Active" is significantly different from zero, seperately for each visit number; The continuous variable of the quality index uses a poisson regression, reporting incidence rate ratios.

Robust standard errors are adjusted for clustering at the neighborhood level.

Missing values across all 3 visits per variable: quality index (9); excellent services (1); ANC facility offers delivery services (4); delivery facility at facility utilized for ANC (4); delivered at ANC facility that offers delivery (4)
